# Recent Advances in Biocatalysis for Drug Synthesis

**DOI:** 10.3390/biomedicines10050964

**Published:** 2022-04-21

**Authors:** Alina Kinner, Philipp Nerke, Regine Siedentop, Till Steinmetz, Thomas Classen, Katrin Rosenthal, Markus Nett, Jörg Pietruszka, Stephan Lütz

**Affiliations:** 1 Chair for Bioprocess Engineering, Department of Biochemical and Chemical Engineering, TU Dortmund University, 44227 Dortmund, Germany; alina.kinner@tu-dortmund.de (A.K.); philipp.nerke@tu-dortmund.de (P.N.); regine.siedentop@tu-dortmund.de (R.S.); katrin.rosenthal@tu-dortmund.de (K.R.); 2 Laboratory for Technical Biology, Department of Biochemical and Chemical Engineering, TU Dortmund University, 44227 Dortmund, Germany; till.steinmetz@tu-dortmund.de (T.S.); markus.nett@tu-dortmund.de (M.N.); 3 Institute of Bio- and Geosciences: Biotechnology (IBG-1), Forschungszentrum Jülich, 52428 Jülich, Germany; t.classen@fz-juelich.de (T.C.); j.pietruszka@fz-juelich.de (J.P.); 4 Institute of Bioorganic Chemistry, Heinrich Heine University Düsseldorf Located at Forschungszentrum Jülich, 52426 Jülich, Germany

**Keywords:** biotransformation, active pharmaceutical ingredient (API), drug discovery, nonribosomal peptide synthesis, protein engineering, scaffold forming, late-stage functionalization, oxyfunctionalization, halogenation, enzyme cascade

## Abstract

Biocatalysis is constantly providing novel options for the synthesis of active pharmaceutical ingredients (APIs). In addition to drug development and manufacturing, biocatalysis also plays a role in drug discovery and can support many active ingredient syntheses at an early stage to build up entire scaffolds in a targeted and preparative manner. Recent progress in recruiting new enzymes by genome mining and screening or adapting their substrate, as well as product scope, by protein engineering has made biocatalysts a competitive tool applied in academic and industrial spheres. This is especially true for the advances in the field of nonribosomal peptide synthesis and enzyme cascades that are expanding the capabilities for the discovery and synthesis of new bioactive compounds via biotransformation. Here we highlight some of the most recent developments to add to the portfolio of biocatalysis with special relevance for the synthesis and late-stage functionalization of APIs, in order to bypass pure chemical processes.

## 1. Introduction

The synthesis of biologically active molecules or active pharmaceutical ingredients (APIs) is challenging. It typically comprises installation and manipulation of stereogenic centers and functional groups (e.g., alcohols, amines). In many cases, the synthesis involves multiple steps and regularly relies on protecting groups. Due to this fact, the production of APIs is usually generating high amounts of waste (*E* factor >100) [1].

Biocatalysis has established itself as an indispensable tool for API synthesis as it offers a number of advantages: Firstly, optimized enzymes as biocatalysts typically deliver superior regio-, stereo-, and enantioselectivity. Secondly, biocatalysis can significantly shorten multi-step synthesis routes by allowing reactions and reaction sequences, which cannot be carried out with classical chemical means. Thirdly, biocatalysis is generally associated with mild reaction conditions, e.g., avoiding the use of toxic reagents and high temperature or pressure [2]. These advantages, in combination, mostly lead to reduced amounts of problematic waste and fewer side products [3]. The advances in protein engineering have enabled a new paradigm: to first conceptually design the desired chemical process and then adapt the biocatalyst to the required reaction conditions [4]. The pharmaceutical industry has been an early adopter of biocatalysis as a tool for API manufacturing. Several biotransformation processes are already applied in the industry, e.g., chiral alcohols using alcohol dehydrogenases, the synthesis of atorvastatin using ketoreductases (KREDs) or sitagliptin production by transaminases (TAs) [5,6]. Mostly, biocatalysis is considered in drug development or manufacturing; however, it can also contribute in earlier stages, even in drug discovery [7].

In this review, we intend to focus on recent advances in implementing biocatalysis for drug synthesis beyond the established reactions. We will mainly focus on expanding the reaction scope by recruiting new enzymes and adapting their substrate scope, as well as the recent trend to combine several enzymes into cascades for API production. For comprehensive reviews on biocatalysis in API synthesis the reader is referred to the existing literature [2,8,9].

## 2. Biocatalysis for Drug Synthesis

### 2.1. Expansion of Product Spectrum

#### 2.1.1. Natural Metabolism as Source of Enzymes and Biochemical Pathways

Primary metabolism maintains all functions of life, including energy balance, growth, reproduction, as well as reaction to internal and external stimuli. However, such conditions are rarely found in ecosystems. Species have to deal with source limitations, abiotic stresses, and biotic interactions ranging from mating to defeating predators. The ability of a species to occupy a particular ecological niche or to adapt to unfavorable conditions is, in many respects, conferred by secondary metabolism. While the variety of primary metabolism pathways is rather limited in terms of numbers, and to some extent general to most species, secondary metabolism is as diverse as the immeasurable number of species themselves. Humans have used natural substances as privileged structures, with regard to their biological activity, more or less consciously for thousands of years, with substances such as spices or colors, but above all as pharmacological agents. However, this use was often limited to extraction from the natural producer and access to effective derivatives was often difficult. Developments in recent decades, such as genome mining, synthetic DNA technologies, and the constructive convergence of chemical and biological methods, are now opening up more intensive and productive access to natural substances, also by enzymological or heterologous means [10].

**Figure 1:**
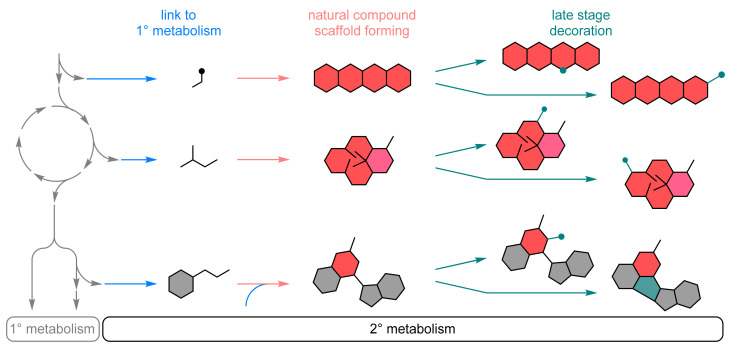
General phases of secondary (2°) metabolism.

Although the number of secondary metabolites is almost immeasurable, the secondary metabolic pathways run in a similar way (Figure 1). The precursor molecules such as acetyl-CoA, amino acids or glycosides are taken from the primary metabolism, and in one enzymatic step or a few steps, the basic structure of the natural substance is formed [11]. For example, a terpene cyclase produces terpene backbones [12], polyketide synthase machinery produces polyketide structures [13] or the phenylpropanoid metabolic pathway starts with the formation of cinnamic acid derivatives by phenylalanine/tyrosine ammonia lyase (PAL/TAL) [14]. Starting from these basic scaffolds, many natural product derivatives are then decorated in vivo by late-stage decorations, such as the introduction of functional groups (oxyfunctionalization, glycosylation, halogenations, etc.) or the conversion of existing functional groups (eliminations, oxidations/reductions, substitutions).

From a synthetic point of view, this scheme might be attractive at any stage: (I) Applying the entire pathway or reprogramming it to produce a particular natural compound and congeners by means of biosynthesis, semisynthesis, and mutasynthesis, respectively. (II) Applying the scaffold key enzyme(s) in a biocatalytic manner to get access to complex natural compounds including sophisticated C–C-couplings or the formation of stereogenic elements [10]. In addition, the last stage, namely the late-stage decorations (III), are attractive to be applied for introducing or modifying functional groups. In particular, the third strategy is instrumental in generating derivate libraries as it is carried out in nature. In contrast to primary metabolism, the enzymes from secondary metabolism are often intentionally promiscuous [15]. Thus, a common task in using the enzymes of secondary metabolism is to narrow down the substrate spectrum, whilst, for enzymes of primary metabolism, the substrate specificity must be altered or widened. Here we highlight some of the most recent developments to add to the portfolio of biocatalytic synthesis of APIs (Table 1).

##### Amide Bond Formation Revisited

Amide bonds are very common structural motifs in many APIs with approximately one quarter of all drugs containing at least one amide bond [26]. Although there exist many established chemical methods to access amide linkages, they often suffer from poor atom efficiency due to the use of activating or coupling reagents. Moreover, toxic or hazardous by-products are commonly generated. Biocatalysis is contributing several new options for accessing amides with relevance for pharmacological use, bypassing the mentioned issues.

**Figure 2:**
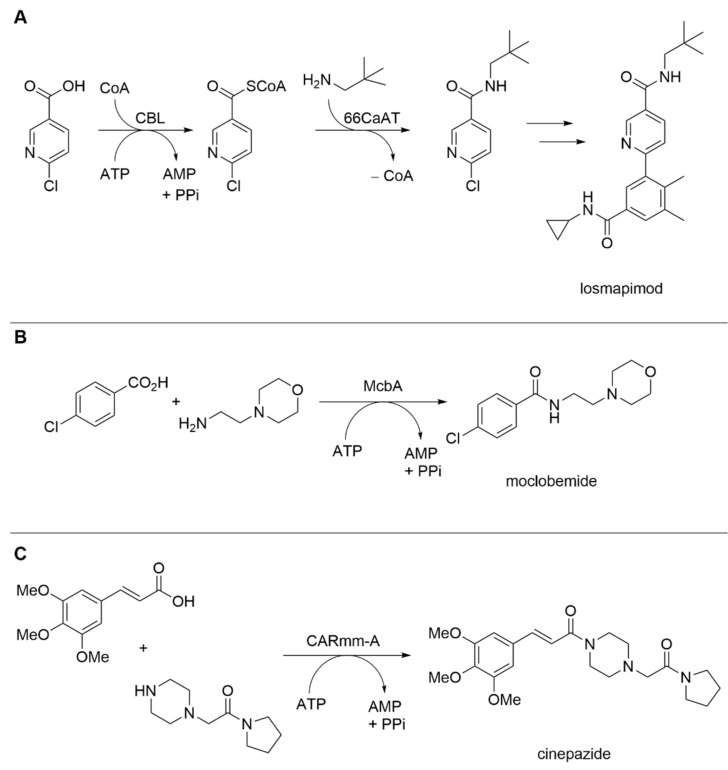
Novel biocatalytic routes to access amides. (**A**) Synthesis of a key intermediate of losmapimod from 6-chloronicotinic acid, and neopentylamine utilizing a 4-chlorobenzoate CL from *Alcaligenes* sp. (CBL), and serotonin hydroxycinnamoyl transferase from *Capsicum annuum* (66CaAT) [16]. (**B**) McbA catalyzed synthesis of moclobemide from *para*-chlorobenzoic acid, and 4-(2-aminoethyl)morpholine [17]. (**C**) Synthesis of cinepazide by the truncated carboxylic acid reductase from *Mycobacterium marinum* CARmm-A [18].

Philpott et al. adopted nature’s way to create amide bonds by combining different ATP-dependent CoA ligases (CLs) with *N*-acetlytransferases (NATs) [16]. Thereby the CL generates a thioester linkage from a corresponding acid and coenzyme A, with consumption of ATP. In a next step, an *N*-acetyltransferase catalyzes the reaction of an amine with the activated thioester to form the amide. Philpott et al. performed an extensive screening of different CLs and NATs and their individual substrate spectra. Combination of the enzymes gave access to a whole variety of amide products. Thereby, a stoichiometric ratio of the carboxylic acid and the amine coupling partner was employed. Synthetic relevance was attested to by production of a key amide intermediate of losmapimod, a selective p38 mitogen-activated protein kinase inhibitor (Figure 2A). Synthesis was achieved utilizing *E. coli* as a whole-cell biocatalyst and exploiting the host’s metabolism for the recycling of cofactors (CoA, ATP). 

ATP-dependent amide bond synthetases (ABSs) are involved in the natural synthesis of secondary metabolites [27]. The ABS McbA from *Marinactinospora thermotolerans* was found to have a broad substrate acceptance for several different amines other than the original substrate, phenylethylamine [28]. Petchey et al. further investigated the substrate tolerance with different β-carboline derivatives and other aryl carboxylic acid acceptors [29]. Surprisingly, McbA accepted naphthoic acid, indole carboxylic acids, as well as benzoic acid as substrates and the synthesis of multiple new products was demonstrated in a preparative scale (50 mg) using 1–5 molar equivalents of 2-phenylethylamine and 2 molar equivalents of ATP, with respect to the acid substrate. These very promising results were complemented by another study, further examining the enzymes substrate scope and implementing an ATP-recycling system [17]. Preparative synthesis of the monoamine oxidase A inhibitor moclobemide was facilitated from *para*-chlorobenzoic acid and 4-(2-aminoethyl)morpholine (Figure 2B). As Petchey et al. [29] solved the crystal structure of McbA, this might also enable future protein engineering to further improve the enzymes features and substrate acceptance.

Lubberink et al. found a way for the biocatalytic selective monoacylation of symmetrical diamines [18]. This is chemically challenging, as one amino group must be selectively protected/deprotected during the course of the synthesis. Wood et al. detected the ability of carboxylic acid reductases (CARs) to catalyze amide bond formations [30]. Lubberink et al. employed a truncated version of a carboxylic acid reductase from *Mycobacterium marinum* denoted CARmm-A [18]. The reaction was optimized by increasing the substrate loading (1, 5, and 10 mM) and the formerly-needed excess of the amine could be lowered from 100 to 17.5 equivalents. To overcome the issue of stoichiometrically-needed ATP, a regeneration system, in the form of a polyphosphate kinase class III from *Cytophaga hutchinsonii*, was successfully employed. With the depicted system in hand, a whole range of products was accessible in a single biocatalytic step, including the APIs lazabemide (anti-Parkinson) and cinepazide (vasodilator) (Figure 2C).

##### Engineered IREDs for Synthesis of Chiral Amines

**Figure 3:**
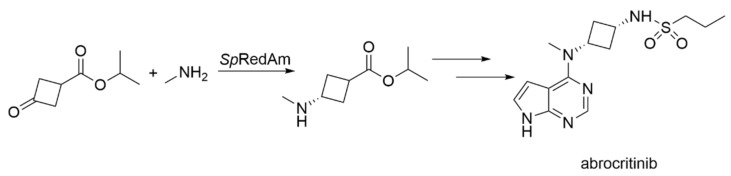
Biocatalytic synthesis of a key intermediate of abrocritinib catalyzed by the engineered reductive aminase *Sp*RedAm from *Streptomyces purpureus* [19].

Imine reductases (IREDs) are a relatively young class of enzymes, having been discovered around the year 2011 [31]. They are NADPH-dependent oxidoreductases, which catalyze the asymmetric reduction of an imine to the corresponding amine. In 2017, a subclass was identified, which is able to efficiently catalyze the direct reductive amination of a ketone to form the asymmetric amine, it was termed reductive aminases (RedAms) [32]. Chiral amines are of high interest for the pharmaceutical industry and thus, IREDs/RedAms were mentioned as an attractive emerging enzyme class in a review from the perspective of the pharmaceutical industry (GlaxoSmithKline–GSK) [33]. Shortly after that, GSK was the first company to enable a kilogram scale production of an API intermediate for the synthesis of the lysine-specific demethylase-1 inhibitor GSK-2879552 utilizing an engineered IRED (Section 2.3) [34]. Recently, the company Pfizer has described another process. In their work, they engineered a reductive aminase for the synthesis of a key intermediate of abrocitinib (Figure 3), a Janus Kinase (JAK) inhibitor, which is a late-stage drug candidate for the treatment of atopic dermatitis [19]. After screening over 80 IREDs from their in-house enzyme library, they discovered *Sp*RedAm from *Streptomyces purpureus* as being the most promising candidate but suffering from a low substrate tolerance. Enzyme engineering was initiated based on the homology model structure and, after only three rounds of engineering, the required criterion of 100 G·L^−1^ substrate loading could be reached. The final enzyme variant *Sp*RedAm-R3-V6 had only four amino acid exchanges N131H/A170C/F180M/G217D and displayed a turnover number (TON) of 36,538, which is an improvement of more than 120-fold in comparison to the wild-type enzyme. The overall enzyme performance was improved more than 200-fold resulting in a final process with a space-time yield (STY) of 60 g·L^−1^·d^−1^ with a purity of >99.5% and great selectivity of >99:1 *cis:trans*. The process was scaled-up to a commercial scale with a batch size of 230 kg. This demonstrates the success biocatalysis can deliver, even in a very limited period and with a relatively young enzyme class.

##### Increasing the Usability of Fe^2+^/α-Ketoglutarate-Dependent Dioxygenases

**Figure 4:**
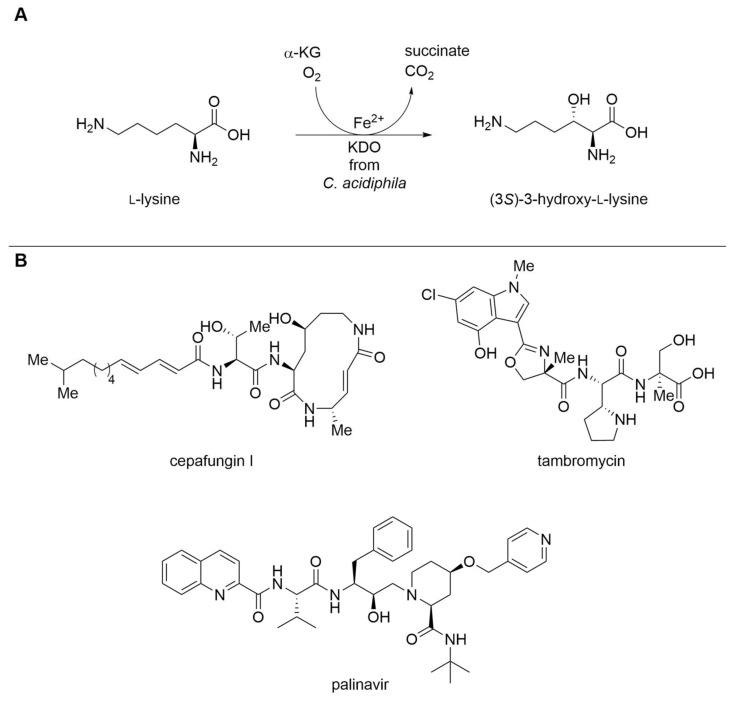
(**A**) Biocatalytic reaction of the KDO from *Catenulispora acidiphila* [39]. (**B**) Molecules of pharmacological interest with chiral hydroxy-l-lysine as building block. α-KG, α-ketoglutarate.

Non-heme Fe^2+^/α-ketoglutarate-dependent dioxygenases (αKGDs) are a large and diverse superfamily of enzymes, capable of catalyzing a plethora of reactions including hydroxylations, halogenations, desaturations, oxidations, and ring formations [35,36]. They all possess a coordinated Fe^2+^ ion and only require α-ketoglutarate and O_2_ as cosubstrates for the enzymatic reaction. The αKGDs accept small and large molecules, depending on the respective enzyme, making them interesting for synthesis, as well as late-stage functionalization. The use of αKGDs in the industrial setting is mainly limited to the derivatization of amino acids and synthesis of antibiotics and has been reviewed recently [37]. Moreover, αKGDs have been employed in a number of chemo-enzymatic syntheses of natural products in the past several years [38]. One of the latest additions to the family of αKGDs are lysine dioxygenases (KDOs), which hydroxylate the amino acid l-lysine with complete chemo-, regio-, and stereospecificity. Depending on the enzyme, different isomers are accessible with hydroxylation of the 3- or 4-position of l-lysine (Figure 4A) [39,40,41]. Enantiopure hydroxy-l-lysines represent valuable intermediates for the synthesis of APIs such as the human immunodeficiency virus (HIV) protease inhibitor palinavir (Figure 4B) [42,43]. KDOs have recently been employed in a combined chemo-enzymatic total synthesis of the potential APIs tambromycin [44] and cepafungin I [45], which are studied for use in cancer treatment (Figure 4B). Hydroxy-l-lysines can also be used as intermediates for the generation of chiral amino alcohols which represent multifunctional chiral building blocks [46]. Rolf et al. demonstrated the identification of novel KDOs utilizing cell-free protein synthesis, which has the potential to drastically speed up the screening of enzymes [47,48]. KDOs have been efficiently used in a whole-cell biocatalyst format for the preparative synthesis of hydroxy-l-lysines with product concentrations of multiple grams per liter [40,48]. Moreover, Seide et al. recently employed KDOs as immobilized enzymes [20]. This led to a remarkable increase in their stability. Immobilized enzymes could be reused in repetitive batches up to seven times, and in preparative biotransformation reactions, 32.4 g·L^−1^ of product were produced, with a specific space-time yield of 100 G·L^−1^·h^−1^ per gram of immobilized enzyme with a KDO from *Catenulispora acidiphila* [20]. Wang et al. demonstrated the first successful engineering of a KDO from *Niastella koreensis* by semi-rationally engineering the active site. The specific activity of the enzyme was improved more than four-fold [43].

**Figure 5:**
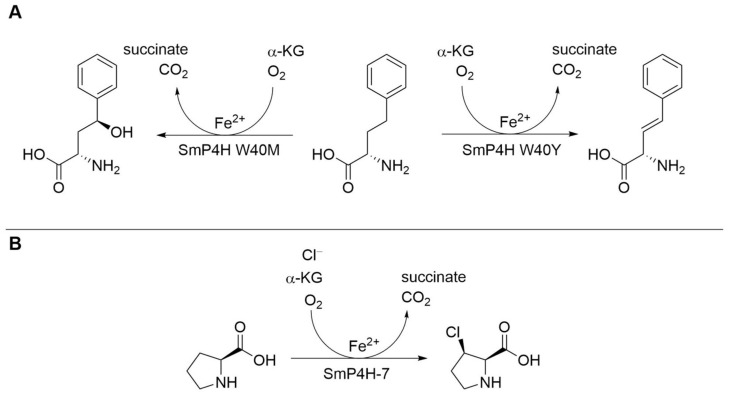
Biocatalytic reactions of the l-proline *cis*-4-hydroxylase SmP4H from *Sinorhizobium meliloti.* (**A**) Engineered enzymatic reaction for hydroxylation or desaturation of l-homophenylalanine [20]. (**B**) Engineered enzymatic reaction with chlorination activity [22].

The group of Buller and coworkers made substantial progress in engineering αKGDs and contributed to understanding the structure–function relationship of the enzymes [21]. They set out to engineer a l-proline *cis*-4-hydroxylase from *Sinorhizobium meliloti* for hydroxylation of a novel substrate, the non-proteinogenic amino acid l-homophenylalanine (Figure 5A) [21]. The respective hydroxylated product represents an intermediate for the synthesis of potential kynureninase or kynurenine monooxygenase inhibitors, which might be of use in the treatment of certain neurodegenerative disorders and cancer. In the study, the W40M/I103L enzyme variant exhibited a >100-fold improved activity and a >300-fold improved catalytic efficiency. Moreover, the turnover number was optimized 10-fold. Interestingly, a single amino acid exchange (W40Y) led to a shift from the hydroxylated product to the desaturated product, showcasing the first αKGD to be engineered from hydroxylase to desaturase activity. In another study, the same L-proline *cis*-4-hydroxylase was engineered for halogenation of the native substrate (Figure 5B) [22]. One amino acid exchange (D108G) led to halogenation activity, which was further elevated over several rounds of enzyme engineering. The final enzyme variant SmP4H-7 showed a 12-fold improvement in the activity and a 98-fold improvement in the catalytic efficiency compared to the starting biocatalyst [22]. The mentioned studies demonstrate the power of enzyme engineering of αKGDs, allowing the conversion of new substrates and even re-programming of the native enzyme reaction.

##### Biocatalytic Scaffold Formation

Many published total syntheses of natural compounds are labeled biomimetic. Certainly, this does not only mean that a certain synthesis step also occurs in a similar way in biosynthesis; rather, the word is also used to denote elegance, effectiveness or even non-conventionality. It is unlikely that a chemist would give the predicate for a profane esterification using a leaving group, although this is also carried out in biosynthesis (e.g., Ac-CoA); it is more likely that something is described as biomimetic when the key step involves the so-called scaffold formation [49,50,51,52]. Scaffold formation, as an early phase of secondary metabolism, not only adds or transforms functional groups but also determines the basic carbon structure of a natural substance (Figure 1). In the past decade, the targeted use of complex scaffold-forming enzymes has made it possible to shorten natural and active agent syntheses and also to make them more precise stereochemically.

**Figure 6:**
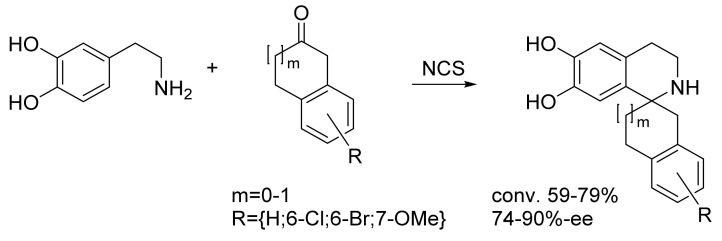
Formation of tetrahydroisoquinolines by Pictet–Spenglerase mutants and the wild-type of the norcoclaurine synthase (NCS) [23].

One example is the synthesis of the tetrahydroisoquinolines scaffold, which is the basis for numerous alkaloids such as quinine, strychnine or vinblastine, and is formed by a Pictet–Spengler reaction between an amine like dopamine and a carbonyl [53,54]. The newly formed *N*-heterocycle moiety is built up by both, a C–N- and a C–C-coupling, including a newly formed stereogenic center. The natural alkaloids are formed from aldehydes. However, Hailes and colleagues developed mutants of the norcoclaurine synthase that also accept ketones or even cyclic ketones, resulting finally in *spiro*-tetrahydroisoquinolines bearing a quaternary stereogenic center (Figure 6) [23]. This complex ring structure is formed within one step with good to moderate yields and the enantiomeric excesses were still sufficiently controlled by these enzyme variants.

**Figure 7:**
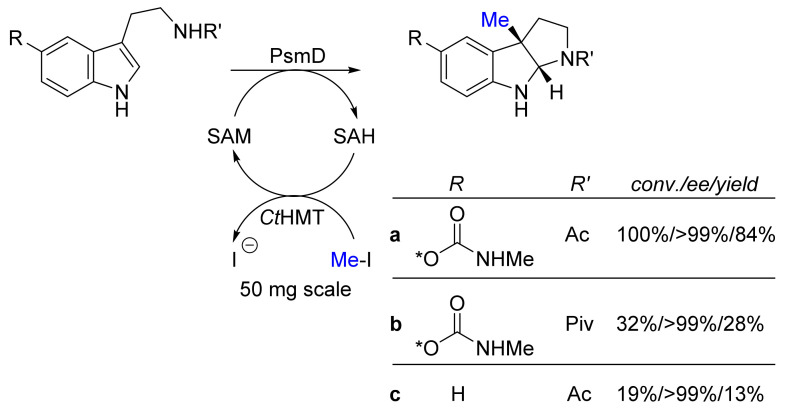
The methylation of the indole causes the cyclisation towards compound. *S*-Adenosyl methionine (SAM) is recycled from *S*-adenosyl homocysteine (SAH) and methyl iodide by the auxiliary enzyme *Ct*HMT [24].

Methylations occur rather often in secondary metabolism, usually as late-stage functionalization. In the case of the physostigmine biosynthesis, the methylation of tryptamine leads to the formation of an additional pyrrolidine ring as a subsequent reaction. The most striking feature here is the formation of a C–C-coupling yielding, simultaneously, a quaternary stereogenic center with high stereoselectivity (Figure 7) [24]. Schneider et al. were able to demonstrate the flexibility of this methylase for various substrates. The methyl group originates from *S*-adenosyl methionine, which is both, unstable and expensive. This drawback for an in vitro reaction could be circumvented by applying a cofactor recycling that employs iodomethane, rather than ATP or other costly co-substrates [55]. This reaction scheme allows for the synthesis of various physostigmine congeners, while physostigmine is known to be an acetylcholine esterase inhibitor [56,57,58].

**Figure 8:**
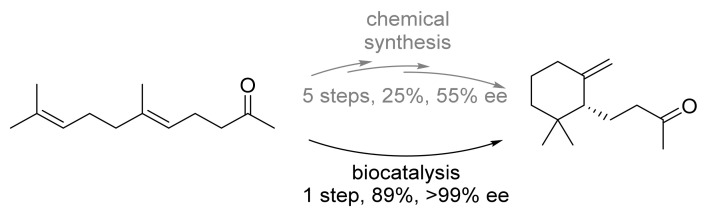
Cyclisation of the ketone to the terpenoid within a single biocatalytic step in contrast to a five-step synthesis [25].

We have already seen, in the case of the norcoclaurine synthase, for the formation of tetrahydroisoquinolines [23], that sometimes adaptions of the enzyme must be made in terms of mutagenesis in order to realize the substrate acceptance. Hauer and colleagues applied the strategy of mutagenesis to channel the substrate formation towards a monocyclic, rather than the bicyclic, product (Figure 8) [25]. Naturally many terpene synthases are rather unselective and generate a bouquet of compounds [15], which can be evolutionary advantageous. *Hauer* and colleagues engineered the active site of the terpene cyclase *Aac*SHC from *Alicyclobacillus acidocaldarius* in a sophisticated, semi-rational manner to exclusively yield the terpenoid compound with a very good yield and an excellent enantiomeric excess. This one-step biocatalytic process has been proven superior in terms of step- and atom-economy in a five-step synthesis, which only yielded a mediocre 25% and 55% *ee*.

These examples impressively show that biocatalysis has emerged to the extent that it can support many active ingredient syntheses at an early stage or bypass pure chemical processes in order to build up entire natural and active ingredient scaffolds in a targeted and preparative manner.

#### 2.1.2. Expansion of Product Spectrum by Engineered Biocatalysts, as Exemplarily Illustrated for Nonribosomal Peptide Synthetases

Many therapeutically relevant peptides, such as the anticancer agent bleomycin, the antibiotic vancomycin or the immunosuppressant cyclosporin A, are biosynthesized by large enzyme complexes, which are composed of nonribosomal peptide synthetases (NRPS) [59]. In recent years, various methods have been developed that allow the rational (re-)programming of these fascinating biocatalysts and, hence, the generation of tailored, bioactive drugs. Before we describe these innovative approaches (Table 2), it is necessary to briefly introduce the thiotemplate-based assembly logic of NRPS.

**Figure 9:**
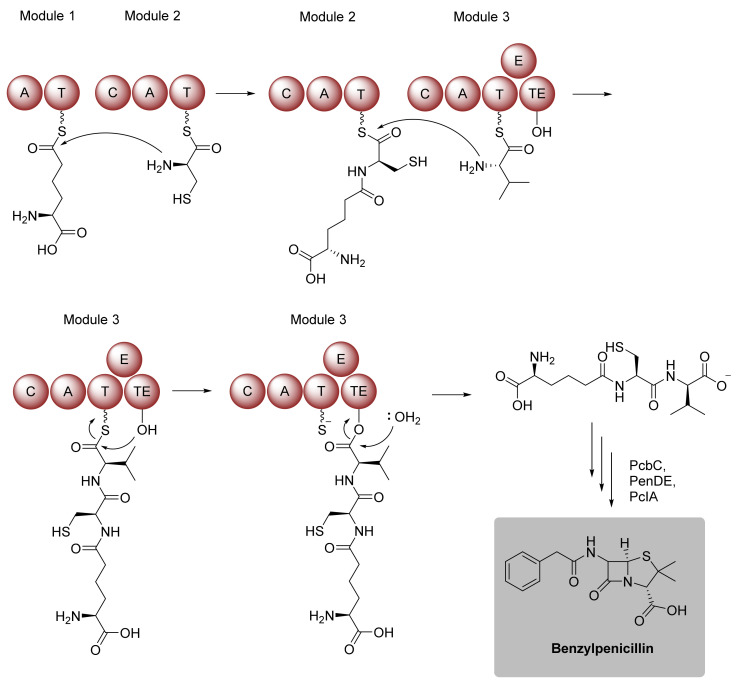
Penicillin biosynthesis in *Penicillium chrysogenum* as textbook example of NRPS assembly logic. Domain notation: A, adenylation; T, thiolation; C, condensation; E, epimerase; TE, thioesterase.

NRPS are modularly organized enzymes that are capable of fusing a variety of amino acid substrates for the generation of peptidic structures. Each module represents a function unit and is solely responsible for the incorporation of one amino acid monomer into the growing peptide chain [60]. For this, a module comprises multiple catalytic domains with different functions. The adenylation (A) domain catalyzes a two-step reaction. After ATP-dependent activation of its substrate, the resulting aminoacyl adenylate is covalently bound to a flexible 4′-phosphopantetheine arm of a neighboring peptidyl carrier protein (PCP) or thiolation (T) domain. A condensation (C) domain mediates the amide linkage between the PCP-tethered substrate and the PCP-bound aminoacyl intermediate of the preceding module [60,61]. This elongation process is consecutively carried out until the terminal NRPS module is reached (Figure 9). Here, a thioesterase (TE) domain detaches the full-length polypeptide from the enzyme complex by hydrolysis or intramolecular lactamization. Some NRPS modules are known to harbor additional domains, which carry out specific modifications such as a methyltransferase (MT) or an epimerase (E) domain [61].

The assembly of peptides by NRPS is more error-prone than ribosomal synthesis, which is due to the substrate flexibility of A domains. In nature, this promiscuity leads to structural diversity within certain families of natural products, when two related amino acids compete for incorporation [62]. Moreover, the substrate flexibility can also be exploited for the generation of natural product derivatives in a process called precursor-directed biosynthesis [63].

**Table 2:** Overview of mentioned nonribosomal peptide synthetases (NRPSs).

Enzyme	Source Organism	Modification	Biocatalyst	Substrate	Product	Process Performance	Reference
NRPS	*Xenorhabdus bovienii*	Artificial splitting of NRPS by inserting natural docking domains	Whole-cell	Amino acids	Xefoampeptides derivatives	Wild-type yield	[64]
NRPS	*Xenorhabdus*; *Photorhabdus*	Introduction of exchange units	Whole-cell	Amino acids	Ambactin derivatives	-	[65,66]
NRPS	*Xenorhabdus*; *Photorhabdus*; *Bacillus*	Introduction of exchange unit condensation domains	Whole-cell	Amino acids	GameXPeptide derivatives	-	[67]
NRPS	*Xenorhabdus* *nematophila*; *Photorhabdus* *luminescens*	Introduction of synthetic zippers	Whole-cell	Amino acids	Xenotetrapeptide; GameXPeptide derivatives	Wild-type yield	[68]
NRPS	*Brevibacillus brevis*	Using zinc fingers as guidance on ssDNA	Isolated enzyme	Amino acids	Gramicidin derivatives	-	[69]
NRPS	*Streptomycetes*	Exchanging FSD that contains key active site residues within A domains	Whole-cell	Amino acids	Enduracididn derivatives	Wild-type yield	[70]

However, the substrate tolerance inherent to NRPS is limited, which is why methods were sought to expand the substrate range of these enzymes. Initial attempts involved the swapping of entire A domains, yet these efforts were met with only limited success. The modest outcome could be attributed to the disruption of interdomain communication and the decreased activity of the engineered NRPS [61]. Furthermore, it has long been assumed that the C domains contribute to substrate proofreading [71]. This presumption stemmed from the observation that substitutions of cognate C–A or A–T–C domain pairs were more successful in NRPS engineering than substitutions of A domains alone. Recently, however, the model of substrate-specifying C domains was challenged both by protein evolution studies [72,73] and by kinetic modeling [74]. It turned out that C domains influence the rate of product formation, but have no direct effect on substrate selection [74]. Clearly, this insight has important implications for NRPS engineering. 

Even under the assumption that A domains act as the sole gatekeepers in NRPS biosynthesis [74], the processivity of the corresponding assembly lines depends, to a large extent, on protein–protein interactions between C, A, and other domains. For this, appropriate recognition or linker sequences are essential. Communication-mediating (COM) domains were initially discovered in 2004, after the construction of NRPS mutants with truncated C-terminal regions [75]. Although a strategy for the biocombinatorial assembly of hybrid NRPS was already described at this time [75,76], it was not until 2018 when the structural basis of the interactions between N- and C-terminal docking domains (DDs) was resolved [77]. In recent years, several studies have demonstrated the feasibility of reprogramming NRPS by swapping or mutating the COM domains and DDs, respectively [77,78,79]. However, it also became clear that this approach, albeit powerful, is limited in its applicability with regard to NRPS enzymes that consist of multiple modules. One solution to this problem was the splitting of multimodule NRPS by natural DD pairs [64].

**Figure 10:**
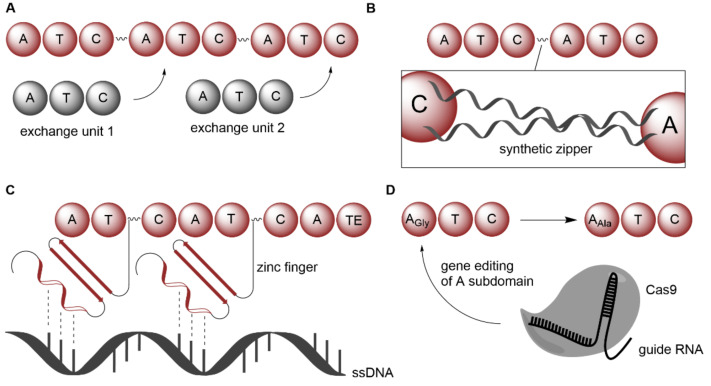
Recently described strategies for the engineering of NRPS. (**A**) Construction of chimeric NRPS from exchange units (XUs). (**B**) Linkage of mono-modular NRPS with synthetic zippers (SZs). (**C**) Generation of DNA-templated NRPS with zinc fingers. (**D**) Swapping of flavodoxin-like subdomains inside A domains with the help of CRISPR-Cas9.

Moreover, alignments of interdomain linker regions from NRPS found in *Photorhabdus* and *Xenorhabdus* bacteria led to the identification of fusion positions between C and A domains that can be exploited for the assembly of customized NRPS [65]. The necessary building blocks are referred to as exchange units (XUs) and consist of three domains (A–T–C or A–T–C/E). By obeying few compatibility rules, the fusion of different XUs leads to functional NRPS (Figure 10A) that produce novel peptides with acceptable yields [65,66]. The combinatorial concept underlying XUs (Figure 10A) could be developed even further after the discovery of a highly conserved fusion point inside the C domains. By merging appropriate NRPS parts at these sites, highly specific and efficient peptide synthetases can be generated [67]. Despite this progress, the construction of chimeric NRPS was still quite laborious. The cloning of the required DNA constructs had to deal with the giant size of NRPS biocatalysts and with repetitive sequence stretches alike. To reduce the complexity of these systems, their splitting into discrete, yet functional subunits was thus desirable. This goal was eventually achieved by furnishing the previously defined XUs with synthetic peptide sequences that can interact with each other via a coiled-coil structural motif [68]. With the help of such synthetic zippers (SZs; Figure 10B), a new type of NRPS architecture has been established that is both productive and easy to handle in the laboratory [68]. An interesting alternative to the use of SZs is to endow split NRPS modules with zinc finger tags [69]. In this way, DNA-templated NRPS can be engineered (Figure 10C), which enables a sequence-controlled biosynthesis of tailored peptides [69].

Except for the replacement of entire domains, modules or XUs, less invasive approaches were also tested, such as the mutagenesis of A domains [80]. An important milestone in this research area was the development of screens for the rapid testing of randomly mutated A domains. The respective assays are based on yeast surface display and enable the selective labeling of catalytically active A domains with fluorophores. In this way, desirable mutations can be readily identified via fluorescence activated cell sorting [81]. 

Another, more targeted strategy is the swapping of the flavodoxin-like subdomain (FSD). The FSD resides within A domains and is known to harbor the key residues for substrate binding [82,83]. Unlike entire A domain swaps, FSD replacements are well tolerated and typically do not interfere with interdomain communication [83]. CRISPR-Cas9 gene editing facilitates the entire procedure (Figure 10D) and also makes the engineering of complex NRPS assembly lines feasible, as recently demonstrated in enduracidin biosynthesis [70]. 

### 2.2. Expanding Biocatalytic Capabilities in Diversification and Late-Stage Functionalization

Late-stage functionalization (LSF) of non-activated carbon centers in natural products and active pharmaceutical ingredients (APIs) is a fundamental tool for drug discovery and drug synthesis [84,85]. However, the direct installation of typically hydroxy-, methyl-, or chloro-groups as well as the formation of C–O, C–N, C–S or C–C bonds in complex scaffolds, challenge synthetic organic chemistry in terms of selectivity and mild reaction conditions to ensure the integrity of sensitive functional groups in drug molecules [86,87]. In addition to metal-catalyzed C–H activation, photocatalysis, or electrosynthesis, biocatalysis has proven to be an advantageous tool for LSFs. High chemo-, regio-, and stereoselectivity, and ambient conditions in aqueous media turned enzyme-catalyzed reactions into promising alternatives to conventional synthesis [86]. As the state-of-the-art native resources do not harbor enzyme-catalyzed equivalents to all synthetic LSFs, identification of novel naturally occurring biocatalysts, in combination with the design of new-to-nature enzymes with desired catalytic activity, are necessary to make biocatalysis more competitive [88]. A selection of current approaches is described in the Table 3.

**Figure 11:**
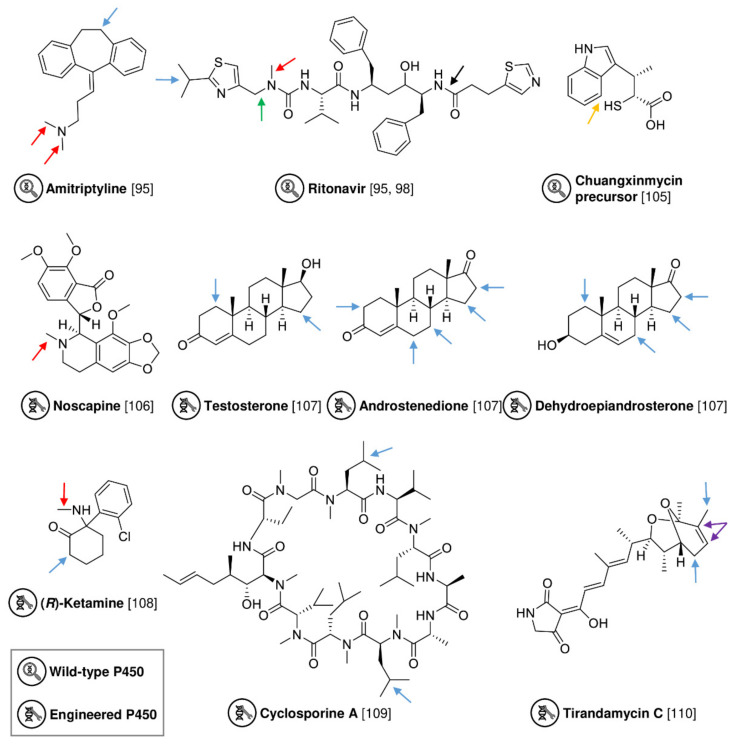
Functionalization of mentioned substrates by wild-type or engineered P450s. Oxidation sites are highlighted with a colored arrow representing hydroxylation (blue), demethylation (red), dealkylation (green), decarbamoylation (black), C-S bond formation (yellow) or epoxidation (purple).

Members of the class of oxidoreductases, such as cytochrome P450 monooxygenases (P450s), laccases, unspecific peroxygenases (UPOs) or lytic polysaccharide monooxygenases, hold great potential for industrial and pharmaceutical applications [89]. To date, the large and versatile group of P450s are the most common biocatalysts for C–H functionalizations [88,90]. These heme enzymes are widely distributed across all domains of life and selectively insert oxygen into various molecules, as well as catalyze heteroatom dealkylations and C–C bond cleavage [91]. Due to their catalytic properties, they have been the subject of latest investigations ranging from genome mining and enzyme activity screening to computation and protein engineering [92]. To broaden the available enzyme portfolio, new P450s were recently identified by genome mining and the transcriptional analysis of wild-type bacteria and fungi (Figure 11) [93,94,95,96,97,98,99]. Schmitz et al. demonstrated the potential of genome mining in combination with strain screening by testing 84 microorganisms for their conversion of seven pharmaceutical compounds (amodiaquine, cyclosporin A, tamoxifen, haloperidol, ritonavir, rapamycin, and testosterone) [94]. They identified three promising strains, which accepted all seven substrates, as well as six additional strains converting six substrates [94]. Further transcriptional analysis and heterologous expression in *E. coli* C42 (DE3) revealed a novel P450, belonging to the CYP105C family, that converts the anti-HIV agent ritonavir to hydroxy ritonavir as the main product (19% yield in the wild-type strain *Actinosynnema mirum* [94]; ~1.6% yield in *E. coli*) [98]. In a two-step screening approach focusing on the discovery and characterization of new P450s, the Urlacher group identified and described the product selectivity of two P450s from *Streptomyces platensis* with a high preference for hydroxylation (CYP105D, 58% hydroxy ritonavir) or demethylation (CYP107Z, 53% demethylated and 7% didemethylated amitriptyline) [95]. However, since the crux of screening is that “you only get what you screen for”, the actual catalytic capability of a strain or an enzyme cannot be displayed in activity screenings with a limited number of substrates. Using a different strategy, the elucidation of biosynthetic pathways for natural products exhibited the involvement of previously unknown P450s, which may be of interest for future investigations [100,101,102,103,104,105]. For instance, unraveling the biosynthesis pathway of the antibiotic chuangxinmycin in *Actinoplanes tsinanensis* led to the identification and characterization of the C-S bond-forming P450 CxnD [105]. 

**Table 3:** Overview of mentioned enzymes catalyzing diversifications and late-stage functionalizations.

Enzyme	Source Organism	Modification	Biocatalyst	Substrate	Product	Process Performance	Reference
CYP105D	*Streptomyces platensis*	Wild-type	*E. coli* cell lysate	Ritonavir	Hydroxy ritonavir	58% conversion	[95]
CYP107Z	*Streptomyces platensis*	Wild-type	*E. coli* cell lysate	Amitriptyline	Demethylated/dide-methylated amitriptyline	53%/7% conversion	[95]
P450 CxnD	*Actinoplanes tsinanensis*	Wild-type	Isolated enzyme (*E. coli*)	Chuangxinmycin precusor	Chuangxinmycin	-	[105]
P450 BM3 variant	*Bacillus megaterium*	Directed evolution	*E. coli* cell lysate	Noscapine	Demethylated noscapine	50% conversion	[106]
P450 BM3 variant	*Bacillus megaterium*	Directed evolution	Isolated enzyme (*E. coli*)	Testosterone	Hydroxy testosterone	≤76% conversion	[107]
CYP154E1 variant	*Thermobifida fusca*	Directed evolution	Whole-cell (*E. coli*)	(*R*)-Ketamine	(2*R*,6*R*)-Hydroxynorketamine	≤85% product	[108]
CYP-sb21	*Sebekia benihana*	Directed evolution	Isolated enzyme (*E. coli*)	Cyclosporin A	Hydroxy cyclosporin A	≤94.6% substrate conversion	[109]
P450 TamI	*Streptomyces* sp. 307-9	Directed evolution	Isolated enzyme (*E. coli*)	Tirandamycin	Hydroxy tirandamycin	New tirandamycin congeners	[110]
*Aae*UPO	*Agrocybe aegerita*	Wild-type	Isolated enzyme	*trans*-Stilbene	4,4′-Dihydroxy-*trans*-stilbene	94% product yield	[111]
*Mro*UPO	*Marasmius rotula*	Wild-type	Isolated enzyme	Clopidogrel	2-Oxo-clopidogrel	46% product	[112]
*Mro*UPO	*Marasmius rotula*	Wild-type	Isolated enzyme	2-Oxo-prasugrel	Active prasugrel metabolite	34% product	[112]
UPO SoLo	*Agrocybe aegerita*	Evolved *Aae*UPO variant	Isolated enzyme	Propranolol	5′-Hydroxypropranolol	15% isolated yield	[113]
UPO JaWa	*Agrocybe aegerita*	Evolved *Aae*UPO variant	Isolated enzyme	Dextromethorphan	*O*-Desmethylnaproxen	82% product	[114]
UPO SoLo-D241G	*Agrocybe aegerita*	Evolved *Aae*UPO variant	Isolated enzyme	Naproxen	Hydroxy tolbutamide	20% product	[114]
*Aae*UPO	*Agrocybe aegerita*	Wild-type	Isolated enzyme	Tolbutamide	4-Hydroxymethyl-tolbutamide	57% product	[114]
Halogenase DklH	*Frankia alni*	Wild-type	Whole-cell (*Streptomyces albus*)	Luteolin	Dichloroluteolin	86% product	[115]
Halogenase WelO5* CA2	*Hapalosiphon welwitschii*	Evolved WelO5* variant	*E. coli* cell lysate	Martinelline-derived substrate	Hydroxylated product	30% isolated yield	[116]
Halogenase WelO5* VLA	*Hapalosiphon welwitschii*	Evolved WelO5* variant	Isolated enzyme	Soraphen A	Mono-chlorinated product	50% conversion	[117]

P450/CYP, cytochrome P450 monooxygenase; UPO, unspecific peroxygenase.

Instead of searching for a native enzyme with the desired catalytic activity, rational protein design and directed evolution are effective tools to get the enzyme of interest (Figure 11) [92]. For more than two decades, numerous approaches of protein engineering have targeted the CYP102A1 (P450 BM3) from*Bacillus megaterium* due to beneficial properties such as its self-sufficiency based on the heme and flavin mononucleotide (FMN)/flavin adenine dinucleotide (FAD)-containing reductase domain on a single polypeptide [118,119,120]. P450 BM3 mutant libraries are often used to identify enzymes that are capable of producing desired drug metabolites and new analogues [92,106,107,121]. For the synthesis of metabolites of the opium poppy alkaloid noscapine, a drug with anticancer activity, site-directed mutagenesis was used to generate a library of 18 variants with mutations located within or close to the active site and the access channel. Five mutations (R47L/A74G/F87A/L188Q/E267V) led to the highly regioselective N-demethylation of noscapine (88% regioselectivity and 50% conversion), whereas no methylation activity could be detected with the wild-type enzyme [106]. In a different approach, site-directed mutagenesis, and especially glycine mutagenesis, have provided access to diverse mono- and dihydroxylated steroids (androstenedione, dehydroepiandrosterone, testosterone) due to altered steroid binding [107]. Almost a decade ago, the Arnold group bioengineered an efficient variant of P450 BM3, P411, by substitution of the conserved axial-ligand cysteine to serine [122]. Further directed evolution resulted in the selective primary amination activity of primary, secondary, and tertiary C−H bonds with yields of up to 93%, which may provide a platform for C-N bond formation of drug molecules [123]. In addition to P450 BM3 and its variants, other P450s have also been selected for protein engineering in recent years, leading to successful selective functionalization of substrates such as the antidepressant (*R*)-ketamine [108], immunosuppressive drug molecule cyclosporin A [109] and natural product tirandamycin [110].

**Figure 12:**
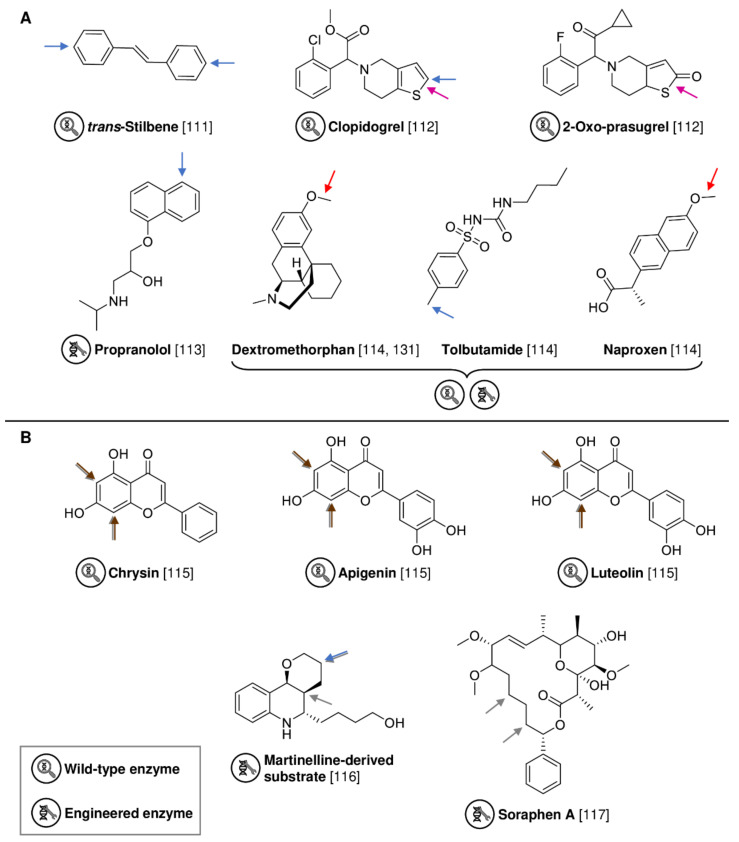
Functionalization of mentioned substrates by wild-type or engineered UPOs (**A**) and halogenases (**B**). Functionalization sites are highlighted with a colored arrow representing hydroxylation (blue), demethylation (red), dealkylation (green), C-S bond cleavage (pink), chlorination (grey) or bromination (brown).

In the last two decades, the exclusively fungal family of robust C-H oxidizing UPOs have emerged as a promising alternative for the oxyfunctionalization of organic substrates [124]. Similar to the “peroxygenase” mode of some P450s, the heme-thiolate enzymes catalyze a broad range of H_2_O_2_-dependent oxidations but do not require NAD(P)H or electron transfer proteins [125]. Because UPO secretion often occurs after 1–3 weeks of fungal cultivation during secondary metabolism, published screening approaches for new wild-type enzymes were mainly performed using heterologous expression rather than the original fungus [126,127]. To date, comprehensive screening data for the functionalization of drug molecules by UPOs are not available, as earlier described, for P450 screenings. However, to bring UPOs out of their infancy and into pharmaceutical application, several groups aimed to elucidate the catalytic properties of both described wild-type UPOs [111,112,128,129] as well as engineered biocatalysts [113,114,130,131] towards relevant bioactive compounds (Figure 12A). Purified model enzymes, *Aae*UPO from *Agrocybe aegerita*, and *Mro*UPO from *Marasmius rotula*, hydroxylated *trans*-stilbene to the resveratrol analogue 4,4′-dihydroxy-trans-stilbene with product yields of 94–96% [111], while high yields were also obtained in the conversion of antithrombotic prodrugs clopidogrel or 2-oxo-prasugrel to bioactive metabolites by both enzymes [112]. Since protein engineering is also a powerful toolbox in UPO research [132], the Alcalde group further evolved JaWa, an evolved variant originated from *Aae*UPO, to SoLo (containing four mutations in the signal peptide and eight in the mature protein) by directed evolution in order to synthesize the human drug metabolite 5′-hydroxypropranolol from the betablocker propranolol on a semi-preparative scale [113]. Furthermore, they could also show that the evolved *Aae*UPO variants PaDa-I, JaWa, SoLo, and SoLo-D241G are suitable biocatalysts with different substrate scopes to synthesize known and novel metabolites of the pharmaceutical agents dextromethorphan, naproxen, and tolbutamide [114]. One major bottleneck of UPO application is its irreversible inactivation at stoichiometric concentrations of H_2_O_2_ [133]. Besides combination with photo- or electrocatalysis, enzyme cascades are a promising strategy to control the in situ supply of H_2_O_2_. Recently, a self-sufficient fusion enzyme, consisting of an evolved aryl-alcohol oxidase from *Pleurotus eryngii* and the SoLo variant, was constructed and successfully used for the synthesis of dextrorphan, a metabolite of dextromethorphan, with a product yield of ~25% [131].

In addition to P450s and UPOs, the strategies of genome mining, enzyme screening and protein engineering have also been successfully applied to other enzyme families. Very recently, a new promiscuous FAD-dependent halogenase, DklH, from *Frankia alni* was identified for the modification of flavonoid compounds by heterologous expression of a biosynthetic secondary metabolite gene cluster in *Streptomyces albus* (Figure 12B) [115,134]. DklH was able to chlorinate, dichlorinate or brominate 14 flavonoids, such as bioactive chrysin, apigenin, and luteolin, with high yields of up to 86% [115]. In a different approach, Buller and colleagues evolved the non-heme iron halogenase WelO5* from *Hapalosiphon* *welwitschia* using structure-guided evolution. Catalytic activity of the engineered WelO5* variant towards chlorination of a promising martinelline-derived substrate, which showed anticancer activity, was efficiently improved with a 290-fold higher total turnover number [116]. Moreover, they demonstrated the potential of algorithm-assisted enzyme evolution by designing a WelO5* variant that is capable of halogenating the bulky acetyl-coenzyme A carboxylase inhibitor soraphen A, a new target of pharmaceutical interest, due to reshaping of the active site [117].

As displayed here, many functionalization reactions catalyzed by enzymes possess great application potential. Limitations, such as substrate solubility [135], co-substrate supply [136], and cofactor regeneration [137], need to be addressed in future work, in which combined approaches such as enzymatic and chemoenzymatic cascades or photobiocatalytic strategies are promising [88,138]. 

### 2.3. Synthetic Enzyme Cascades

**Figure 13:**
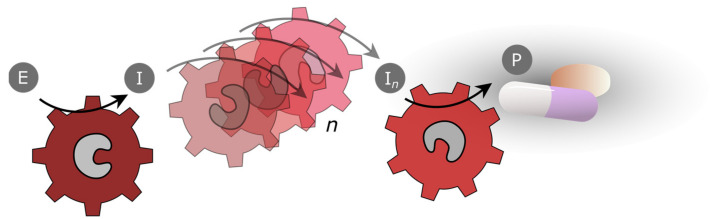
Schematic representation of an enzyme cascade for drug synthesis. Two plus n enzymes can participate in product production (*n* ≥ 0). E, educt; I, intermediate; P, product.

Along with the intriguing properties of enzymes, with their high specificity and selectivity, efficiency under mild reaction conditions, and sustainable catalysis, several reaction steps using various isolated enzymes can also be combined in one pot. Since enzymes mostly prefer similar reaction conditions such as an aqueous buffer and physiological pH and temperatures, it is suitable to catalyze their reactions simultaneously in the same reactor (Figure 13). The additional advantages of such multi-enzymatic reactions, also called enzyme cascades, include the reduced demand for intermediate isolation, which decreases the need for large amounts of solvents, the equilibrium-shift towards the product side in these systems, and complex molecules can be built up with simple starting materials [139]. This is especially interesting for drug synthesis, since the compound structures are often complex and the usage ofbulk materials as educts reduces the costs of their production. Furthermore, using enzyme cascades gives the advantage of being able to design the reaction steps to a customized and efficient synthesis route. However, several parameters, such as reaction conditions or inhibitory effects, can influence the interplay of the cascade components. Therefore, the complex systems often suffer from low performances at the beginning and optimization effort has to be put in [140]. By engineering the biocatalysts, the adjustment towards tailored properties enables both precising and optimizing of the specificities of the enzyme thus enlarging the reaction scope even further. To tackle the challenging task of creating a functional multi-enzymatic pathway for API synthesis and improving the performance, several approaches have been published, ranging from bioretrosynthetic to chemoenzymatic strategies [141,142]. Numerous examples exist for the impressive synthetic possibilities for drugs enabled by enzyme cascades, some of the most recent of which are presented here (Table 4).

**Figure 14:**
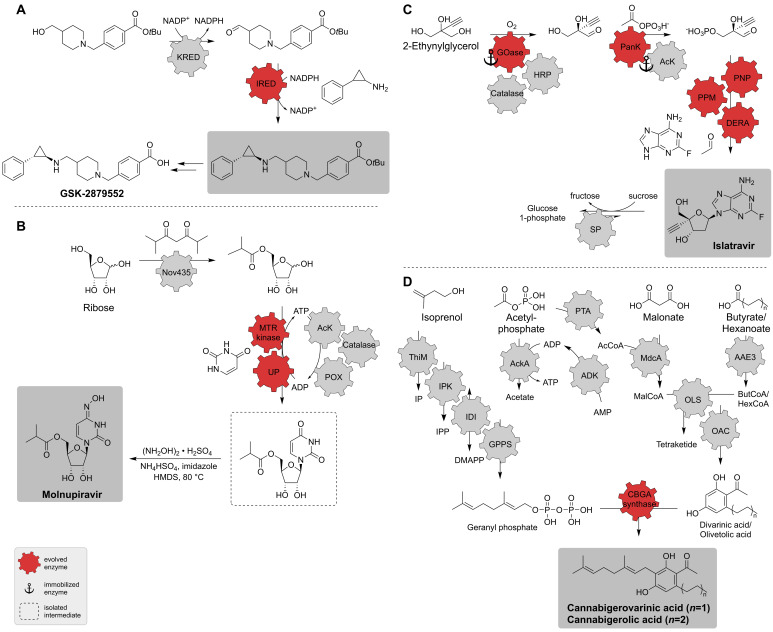
(**A**) Enzyme cascades for the synthesis of GSK-2879552 [34], (**B**) Molnupiravir [149], (**C**) Islatravir [141], and (**D**) cannabigerovarinic acid and cannabigerolic acid (CBGA) [152]. AAE3, acyl activating enzyme 3; AcK, acetate kinase; ADK, adenylate kinase; DERA, deoxyribose 5-phosphate aldolase; GOase, galactose oxidase; GPPS, geranyl pyrophosphate synthase; HRP, horseradish peroxidase; IDI, isopentenyl diphosphate isomerase; IPK, isopentenyl kinase; IRED, imine reductase; KRED, ketoreductase; MdcA, malonate decarboxylase α subunit; MTR kinase, 5-S-methylthioribose kinase; Nov435, Novozym 435; OAC, olivetolic acid cyclase; OLS, olivetol synthase; PanK, pantothenate kinase; PNP, purine nucleoside phosphorylase; POX, pyruvate kinase; PPM, phosphopentomutase; PTA, phosphotransacetylase; SP, sucrose phosphorylase; ThiM, hydroxyethylthiazole kinase; UP, uridine phosphorylase.

As mentioned, IREDs are exiting enzymes for the insertion of chiral amines. Reactions resulting in these groups are, for example, reductive aminations, which in turn can be catalyzed by some IREDs with a carbonyl group and an amine as substrates. Such an enzyme was utilized in the two-enzyme cascade for the synthesis of the investigational drug GSK-2879552 (Figure 14A) [34]. GSK-2879552 is a lysine-specific histone demethylase-1A (LSD1 or lysine demethylase 1A, KDM1A) inhibitor and was investigated for its activity against small cell lung cancer (SCLC), acute myeloid leukemia (AML), and myelodysplastic syndrome (MDS), however clinical trials were terminated [143,144]. In 2019, GlaxoSmithKline (GSK) published an enzyme cascade for the production of the protected precursor of GSK-2879552. For the synthesis of this intermediate, a suitable IRED was found that was even able to convert its substrate, a racemic *trans* amine compound with the stereogenic centers already included, with excellent enantioselectivity. To further improve its usability for a commercially viable process, the enzyme was evolved for better pH tolerance, increased substrate concentrations and product yield, and reduced biocatalyst loading. After three rounds of evolution, a 38,000-fold improvement in the turnover number (TON) was reached and the other criteria were either met or exceeded. A KRED was then implemented to receive the aldehyde substrate from its corresponding alcohol in situ. Furthermore, this enzyme allowed the recycling of the cofactor nicotinamide adenine dinucleotide phosphate(H) (NADP(H)) in a hydrogen borrowing manner resulting in a redox-neutral cascade. GSK-2879552 precursor production was run at a 5 g scale with a 48.3% yield, 99.5% *ee* and 97.9% purity [34].

Metaraminol, an API used to treat hypotension during anesthesia [145], also comprises a chiral amine [146]. The synthesis from 3-hydroxybenzaldehyde (3-OH-BA) and *L*-alanine as an amine donor is catalyzed by a pyruvate decarboxylase (PDC) and a transaminase (TA). Often, TAs have unfavorable equilibria for transamination reactions and the challenge is to shift it towards the product side. Mack et al. exploited the solubility of metaraminol in organic solvents to pull the equilibrium by the in situ product removal (ISPR) [146]. To include liquid–liquid extraction, a suitable organic solvent was sought, measured on transaminase activity and conversion. With additional optimization of the pH for the reaction, an increased yield of 29%, compared to a 14% conversion, could be achieved [146].

Other enzyme cascades were developed to synthesize relevant pharmaceutical products such as molnupiravir (MK-4482, formerly EIDD-2801) from simple starting materials. Molnupiravir is a compound which is currently investigated for the treatment of the coronavirus disease 2019 (COVID-19) with promising results during clinical trials to reduce the risk of hospitalization [147]. Due to the urgent need of the API during the pandemic, there was considerable interest in the development of synthetic routes. Chemical routes suffer from drawbacks such as low yields and harsh reaction conditions, which is why biocatalytic alternatives were sought. To introduce the *N*-hydroxy group at the base, the enzyme cytidine deaminase was engineered to transform cytidine to *N*-hydroxy-cytidine at high concentrations, with high conversion and low biocatalyst loadings [148]. The resulting intermediate could be further acylated to the final product by the enzyme Novozym^®^ 435, an enzyme that is used in an enzyme cascade developed by Merck & Co in 2021 (Figure 14B) [149]. This route was designed rationally and enzymes were sought to catalyze the necessary reactions to use the simple starting materials ribose and uracil. The first enzyme, Novozym^®^ 435, acylates the primary alcohol of ribose at the 5′ position. Afterwards, 5-*S*-methylthioribose (MTR) kinase first activates the anomeric center at the 1′ position by phosphorylation and then uridine phosphorylase (UP) couples uracil to this position. The MTR kinase and UP were evolved for improved activity on the non-natural substrates leading to an 80- and a 100-fold improvement. In addition, an ATP regeneration system consisting of pyruvate oxidase, acetate kinase, and a catalase was included to shift the equilibrium towards the product. The resulting molnupiravir precursor was isolated and finalized by a chemical step to convert the amidic carbonyl of uracil to an oxime. Molnupiravir was gained with a 69% overall yield and notably, the whole cascade with enzyme discovery and evolution, as well as the reaction development and implementation on a large scale, was developed in only 6 months [149]. Furthermore, the patented chemical synthesis consisting of 10 steps yields molnupiravir in much lower overall yields, at around <10% [150].

**Table 4:** Overview of mentioned enzyme cascades.

Enzyme	Source Organism	Modification	Biocatalyst	Substrate	Product	Process Performance	Reference
IRED; KRED	Diverse	Directed evolution (one enzyme)	Isolated enzymes	Amine tranylcypromine sulfate, alcohol precursor	Protected GSK-2879552	5 g scale with 48.3% yield, 99.5%-*ee,* 97.9% purity	[34]
Nov435; MTR kinase; UP; AcK; catalase; POX	Diverse	Directed evolution (two enzymes)	Isolated enzymes	Ribose, uracil	Molnupiravir	69% overall yield	[149]
GOase; HRP; catalase; PanK; AcK; PNP; PPM; DERA; SP	Diverse	Directed evolution (five enzymes)	Isolated enzymes	2-Ethynylglycerol	Islatravir	51% overall yield	[141]
ThiM; IPK; IDI; GPPS; ADK; AcKA; PTA; MdcA; AAE3; OLS; OAC; CBGA synthase	Diverse	One engineered enzyme by Rosetta design [151]	Isolated enzymes	Isoprenol, acetylphosphate, malonate, butyrate/hexanoate	Cannabigerolic acid (CBGA); cannabigerovarinic acid (CBGVA)	480 mg L^−1^ (CBGA), 580 mg L^−1^ (CBGVA)	[152]
ADK; 2 PPK2; cGAS	Diverse	-	Isolated enzymes	Adenosine, GTP	2′3′-cGAMP	0.08 mol per mol adenosine	[153]
GK; AK; AcK; cGAS	Diverse	Directed evolution (four enzymes)	Isolated enzymes	Nucleotide monothiophosphates	MK-1454	62% isolated yield	[154]

AAE3, acyl activating enzyme 3; AcK, acetate kinase; ADK, adenosine kinase; ADK, adenylate kinase; AK, adenylate kinase; cGAS, cyclic GMP-AMP synthase; DERA, deoxyribose 5-phosphate aldolase; GK, guanylate kinase; GOase, galactose oxidase; GPPS, geranyl pyrophosphate synthase; HRP, horseradish peroxidase; IDI, isopentenyl diphosphate isomerase; IPK, isopentenyl kinase; IRED, imine reductase; KRED, ketoreductase; MdcA, malonate decarboxylase α subunit; MTR kinase, 5-S-methylthioribose kinase; Nov435, Novozym 435; NRPS, nonribosomal peptide synthetase; OAC, olivetolic acid cyclase; OLS, olivetol synthase; PanK, pantothenate kinase; PNP, purine nucleoside phosphorylase; POX, pyruvate kinase; PPK2, polyphosphate kinase 2; PPM, phosphopentomutase; PTA, phosphotransacetylase; SP, sucrose phosphorylase; ThiM, hydroxyethylthiazole kinase; UP, uridine phosphorylase.

Besides this forward approach for pathway design, an imposing synthesis route for islatravir was developed using a bioretrosynthetic method by Merck & Co. and Codexis in 2019 (Figure 14C) [141]. Islatravir was in clinical trials for the treatment of HIV but the study was paused in 2021 due to the reduction of important immune cells [155]. For the synthetic route, they used the bacterial nucleoside salvage pathway as a scheme. With this scheme, they chose the reactions and substrates needed to build the nucleoside analog, starting at the product [141]. The reactions, in reversed order, are the attachment of the nucleobase, the phosphorylation at this position, and the assembly of the deoxyribose out of a glyceraldehyde 3-phosphate derivative and acetaldehyde. The enzymes purine nucleoside phosphorylase (PNP), phosphopentomutase (PPM), and deoxyribose 5-phosphate aldolase (DERA) were suitable for these reactions, respectively. The glyceraldehyde 3-phosphate derivative was obtained from the cascades starting point 2-ethylglycerol through oxidation and phosphorylation by pantothenate kinase (PanK) and the correct stereocenter was introduced by galactose oxidase (GOase). The enzymes of this main pathway were engineered by directed evolution to meet certain criteria like increased activity, the acceptance of non-natural substrates or high substrate concentrations and stereoselectivity. Four further enzymes were included, namely horseradish peroxidase (HRP), catalase, acetate kinase (AcK) and sucrose phosphorylase (SP) for, e.g., cofactor regeneration or an equilibrium shift towards islatravir by removing the released phosphate during the last step. In addition, some biocatalysts of the nine-enzyme cascade were immobilized for simpler product isolation. Finally, islatravir was received with a 51% overall yield without any isolation steps out of simple achiral starting materials [141]. 

Along with these rather rational approaches for the design of synthetic routes, natural pathways can be used as template as well. The in vitro enzymatic syntheses of the cannabinoids cannabigerolic acid (CBGA) and cannabigerovarinic acid (CBGVA) were realized by following and modifying the glycolysis and mevalonate pathway towards geranyl pyrophosphate (GPP) [151]. GPP is used for the prenylation of fed aromatic substrates leading to the cannabinoids yielding titers over 1 g L^−1^. Cannabinoids have interesting pharmaceutical properties and tetrahydrocannabinol (THC) and cannabidiol (CBD) are already approved drugs, though, many more clinical trials investigate other cannabinoids for the treatment of various diseases as well [156]. However, this large cascade consists of 25 enzymes, lowering the commercial viability. In 2020, the system was improved to synthesize CBGA and CBGVA by twelve enzymes, also allowing the production of the aromatic precursors in situ (Figure 14D) [152]. This one-pot cascade can be separated into four modules. The precursors GPP and olivetolic acid (OA) or divarinic acid (DA) have to be built up by two separate routes with four and five enzymes, respectively. The synthesis of GPP follows a simplified isoprenoid biosynthesis pathway starting with isoprenol. OA or DA are built up from malonate and butyrate or hexanoate. The need for ATP, an important cofactor for the cannabinoid synthesis, could be reduced by three equivalents, by clever pathway adjustment using a non-natural reaction of the malonate decarboxylase (MdcA) α subunit and a further module regenerates ATP from acetyl-phosphate. The final step of the system prenylates OA or DA to the cannabinoid product by CBGA synthase. Remarkably, the system produced CBGA and CBGVA in the ~0.5 g L^−1^ range in 10 h, which is nearly two orders of magnitude higher than by using an engineered yeast, where the highest reported titer was 8 mg L^−1^ [152,157].

**Figure 15:**
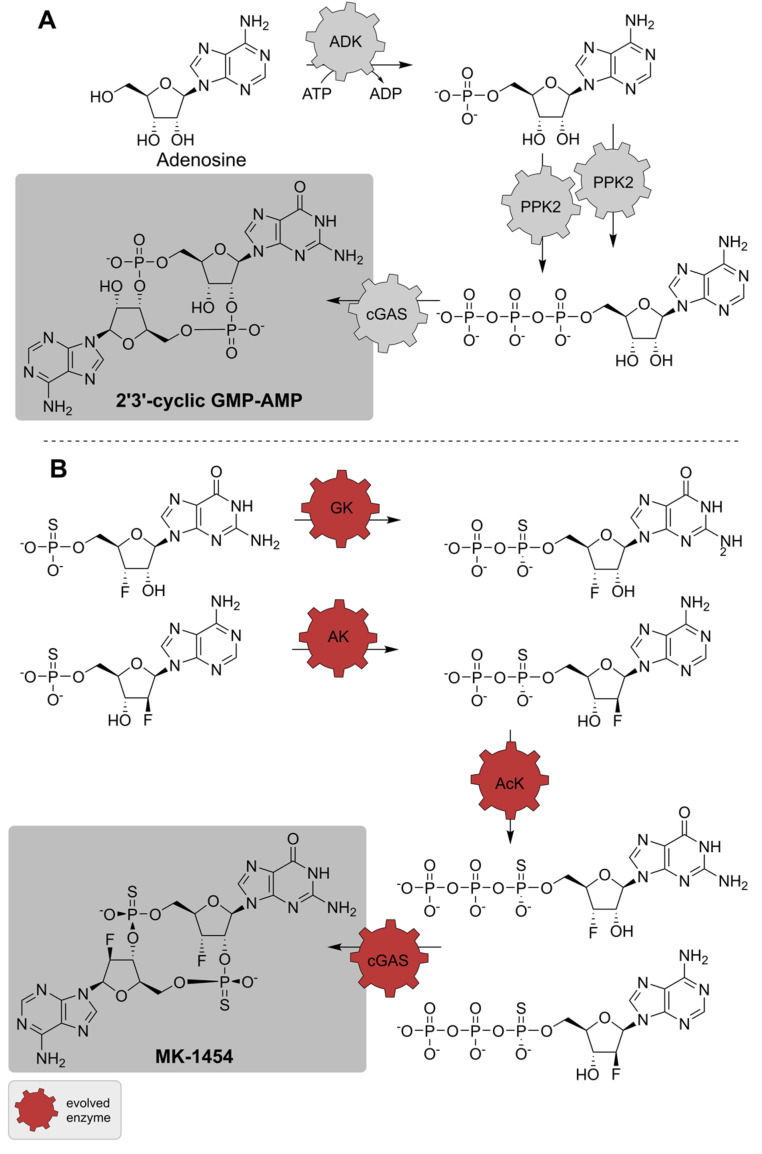
(**A**) Multi-enzymatic reactions for the synthesis of 2′3′-cyclic GMP-AMP [153], and (**B**) MK-1454 [154]. AcK, acetate kinase; ADK, adenosine kinase; AK, adenylate kinase; cGAS, cyclic GMP-AMP synthase; GK, guanylate kinase; PPK2, polyphosphate kinase 2.

Another example for the ability of biocatalysis to shorten synthesis routes is the generation of cyclic dinucleotides (CDNs). CDNs are ubiquitously occurring second messenger molecules. In vertebrates, the cyclic GMP-AMP dinucleotide 2′3′-cGAMP induces the production of interferons and cytokines as part of the innate immune system by binding to the STING receptor [158]. This makes 2′3′-cGAMP a promising candidate for pharmaceutical applications as potent immune-oncology therapeutics. Some derivatives are currently investigated for their activity against cancer and as vaccine adjuvants [159]. While the chemical synthesis of cyclic dinucleotides is possible, it requires multiple reaction steps, complex protective group chemistry and it suffers from low yields [160]. Rosenthal et al. demonstrated the production of several 2′3′-cGAMP derivatives in one reaction step using a truncated version of human cyclic GMP-AMP synthase (cGAS), exploiting the enzymes natural substrate promiscuity [161]. Thereby, 2′3′-cGAMP, cyclic GMP-2′-F-AMP and cyclic GMP-8-NH_2_-AMP were produced in a preparative scale. The fluorine derivative was shown to confer improved STING binding, attesting its pharmacological relevance [162]. To further optimize the synthesis of CDNs and to lower the substrate costs, enzymes were implemented to deliver a suitable substrate *in situ*. In this regard, Becker *et al*. developed an enzyme cascade to produce the substrate ATP for a single-step transformation by cGAS to form the CDN 2′3′-cGAMP. The multi-enzymatic reaction was developed to form ATP by phosphorylating adenosine, which is subsequently cyclized with GTP to synthesize the cyclic dinucleotide (Figure 15) [153]. In the first step of the cascade, adenosine kinase (ADK) phosphorylates the nucleoside to AMP by consuming ATP. AMP is then converted stepwise to ATP by two homologues of polyphosphate kinase 2 (PPK2). This enables the recycling of the formed ADP by the ADK and the chosen PPK2s allows the usage of cheap polyphosphate (polyP) as the substrate. The formed nucleotide triphosphate serves with GTP as the substrate cGAS, which produces 2′3′-cGAMP. To increase synthesis rates, yield, and enzyme usage, substrate concentrations, as well as the ratios of the enzyme concentrations, were adjusted according to their specific activities. Several attempts were conducted to find the best composition. The 2′3′-cGAMP production could be increased ~2,5-fold and ultimately, the product was gained by the four-enzyme cascade with 0.08 mol per mol adenosine, which is in the same range as the chemical synthesis [153]. Recently, Merck & Co and Codexis published the multi-enzymatic synthesis of the derivative ML-1454 (Ulevostinag), a more potent fluorinated phosphorothioate CDN analogue [154]. The cyclization of thioGTP and thioATP was also performed by cGAS, though, the enzyme was evolved towards improved reactivity in seven rounds. The challenge to form the correct CDN diastereomer with achiral thiophosphategroups was solved by an enzymatic desymmetrization. Thiomonophosphates were phosphorylated by adenylate kinase (AK) or guanylate kinase (GK) and the resulting thiodiphosphates were subsequently stereoselectively phosphorylated by an AcK. AK, GK, and AcK were evolved in 2–5 rounds, leading to good diastereomeric ratios and high yields. The merging of the enzymatic reactions to a one-pot cascade resulted in a 62% isolated yield from nucleotide monothiophosphates [154]. This is a remarkable production, especially in comparison to chemical synthesis, where yields around 5% for 2′3′-cGAMP within 3 days are achieved [160,163]. In addition, these examples highlight the difficulties of using enzyme cascades. The composition of the reaction, such as enzyme ratios and substrate concentrations, plays a vital role for the performance of the systems. Engineering the enzymes towards tailored properties can improve the interplay of the catalysts but it is time- and cost-intensive.

Chemoenzymatic cascades combine the advantages of biocatalysis with classical chemistry to broaden the reaction scope. However, mutual inactivation is a problem in these synthesis approaches since enzymes have to tolerate harsher conditions such as solvents [164] and higher temperatures, and chemical catalysts can be inhibited in aqueous solutions as well. Nonetheless, lipases and serine proteases were among the first enzymes successfully employed in such syntheses [165]. However, few examples exist for the simultaneous usage of several enzymes and chemical conversions in one pot for drug synthesis. Here, the production of (*S*,*S*)-sertraline is shown, which uses two enzymes and a chemical reaction, in separately performed steps [142]. Sertraline is the API in Zoloft^®^ (Pfizer), used for the treatment of depression and anxiety [166,167]. Enzymatic steps were integrated to receive a stereoselective keto-precursor of sertraline [142]. For this purpose, the racemic mixture of this keto-precursor was selectively reduced by a KRED and subsequently re-oxidized by either a chemical step or a by a laccase to get the enantiopure keto-educt. Suitable KRED and reaction conditions were selected by the screening of enzyme variants and temperature, pH, and racemic substrate concentrations. Finally, the enantiopure alcohol-precursor was gained with a >99% *ee* and 29% conversion after 7 h. The final amination was performed with a chemical step, since a biocatalytic amination of the racemic educt by IREDs or TAs were unsuccessful. (*S*,*S*)-sertraline was obtained with a >99% *ee* and an overall yield of 16% [142].

Several approaches for the challenging synthesis of drugs by multi-enzymatic reactions were developed, including the rational and bioretrosynthetic building of the reaction route, creation of enantioselective products by specialized enzymes, and performance improvement by adjusted enzyme ratios or equilibrium shifts by ISPR. Partly, product titers were higher using the enzyme cascades than in cell-based productions. These examples demonstrate the broad versatility and applicability of enzyme cascades for API synthesis and as more research is put to this topic, more examples and even industrial applications will be the outcome.

However, the development of new processes not only includes the development of the synthesis step, but also an integrated view of upstream and downstream. It is usually not the synthesis step that incurs the highest costs, but the downstream process. Nevertheless, the higher the titer and space–time yield and the fewer the by-products in the synthesis step, the easier the downstream processing. Therefore, the development and optimization of the most efficient biocatalysis possible will continue to be of concern in the future. 

## 3. Conclusion and Outlook

Recent progress in protein engineering and adoption of this technology by several pharmaceutical companies has allowed even more enzymes to be made available for production, tuning their properties accordingly. Several examples and recently made progress in the field of biocatalytic production of APIs have been discussed in this review. The potential of biocatalysis for drug synthesis, however, is far from being exhausted. New biocatalysts can be discovered by genome mining and screening [94]. Recent research is moving towards de novo enzyme design, which is certainly a tool that will be utilized even more in the future [168,169,170,171,172]. Once discovered, new biocatalysts can be further optimized or adopted for specific reactivities using enzyme engineering tools [173,174,175]. Given the vast protein sequence space and the ever-increasing tools to explore it, it is safe to assume that more enzymes and enzyme cascades will be utilized for drug synthesis in the future.

## Figures and Tables

**Table 1:** Overview of mentioned enzymes from the natural metabolism.

Enzyme	Source Organism	Modification	Biocatalyst	Substrate	Product	Process Performance	Reference
4-Chlorobenzoate ATP-dependent CoA ligase (CBL) and serotonin hydroxycinnamoyltransferase (66CaAT)	*Alcaligenes* sp.; *Capsicum annuum*	-	Whole-cell	6-Chloronicotinic acid; neopentylamine	6-Chloro-*N*-neopentylnicotinamide (losmapimod key intermediate)	83% conversion, 74% isolated yield	[16]
ATP-dependent amide bond synthetase McbA	*Marinactinospora thermotolerans*	-	Isolated enzyme	4-Chlorobenzoic acid; 4-(2-aminoethyl) morpholine	Moclobemide	70% conversion, 64% isolated yield	[17]
Carboxylic acidreductase CAR*mm*-A	*Mycobacterium marinum*	Truncated enzyme variant	Isolated enzyme	3,4,5-trimethoxycinnamic acid; piperazine acetic acid pyrrolidine	Cinepazide	18% isolated yield	[18]
*Sp*RedAm-R3-V6	*Streptomyces purpureus*	Engineered variant (four amino acid exchanges)	Isolated enzyme	Isopropyl 3-oxocyclobutane-1-carboxylate; monomethylamine	Isopropyl 3-(methylamino) cyclobutane-1-carboxylate (abrocritinib key intermediate)	60 g L^−1^ d^−1^, purity >99.5%, selectivity >99:1 *cis:trans*	[19]
Lysine dioxygenase (KDO)	*Catenulispora acidiphila*	Immobilization with HaloTag^®^	Isolated enzyme	l-Lysine	(3*S*)-3-Hydroxy- l-lysine	32.4 g L^−1^, 100 g L^−1^ h^−1^ per g_immobilized enzyme_	[20]
Proline hydroxylase SmP4H	*Sinorhizobium meliloti*	Engineered variant (two amino acid exchanges)	Isolated enzyme	l-Homophenylalanine	γ-Hydroxylated l-homophenylalanine	k_cat_ 1.680 ± 0.068 min^−1^, 35.3% yield	[21]
Proline hydroxylase SmP4H	*Sinorhizobium meliloti*	One amino acid exchange (W40Y)	Isolated enzyme	l-Homophenylalanine	3,4-Desaturated l-homophenylalanine	k_cat_ 0.83 ± 0.02 min^−1^, 50.7% yield	[21]
Proline hydroxylase SmP4H	*Sinorhizobium meliloti*	Engineered variant (six amino acid exchanges)	Cell lysate	l-Proline	*cis*-3-Chloro- l-proline	4.86 ± 0.16% yield, >98.5%-*ee*	[22]
Norcoclaurine synthase (*tf*NCS)	*Thalictrum flavum*	Directed evolution	Cell lysate	Aromatic β-amine; ketone	Tetrahydroisoquinolines	59-79% conversion 74-90%-*ee*	[23]
Methyl transferase PsmD; halide methyl transferase (*ct*HMT)	*Streptomyces* *griseofuscus*; *Chloracidobacterium* *thermophilum*	-	Isolated enzymes including SAM- cofactor recycling	Tryptamines	Physostigmines	13-84% isolated yield >99%-*ee*	[24]
Terpene cyclase (*Aac*SHC)	*Alicyclobacillus acidocaldarius*	Semi-rational design	Isolated enzyme	Geranyl acetone	γ-Dihydroionone	89% isolated yield, >99%-*ee*	[25]
